# A Conditional Process Model to Explain Somatization During Coronavirus Disease 2019 Epidemic: The Interaction Among Resilience, Perceived Stress, and Sex

**DOI:** 10.3389/fpsyg.2021.633433

**Published:** 2021-05-20

**Authors:** Fangfang Shangguan, Chenhao Zhou, Wei Qian, Chen Zhang, Zhengkui Liu, Xiang Yang Zhang

**Affiliations:** ^1^Beijing Key Laboratory of Learning and Cognition, School of Psychology, Capital Normal University, Beijing, China; ^2^CAS Key Laboratory of Mental Health, Institute of Psychology, Chinese Academy of Sciences, Beijing, China; ^3^Department of Psychology, University of Chinese Academy of Sciences, Beijing, China

**Keywords:** resilience (psychological), perceived stress, somatic symptom, somatization, gender, conditional process analysis

## Abstract

**Background:**

More than 15% of Chinese respondents reported somatic symptoms in the last week of January 2020. Promoting resilience is a possible target in crisis intervention that can alleviate somatization.

**Objectives:**

This study aims to investigate the relationship between resilience and somatization, as well as the underlying possible mediating and moderating mechanism, in a large sample of Chinese participants receiving a crisis intervention during the coronavirus disease 2019 epidemic.

**Methods:**

Participants were invited online to complete demographic information and questionnaires. The Symptom Checklist-90 somatization subscale, 10-item Connor–Davidson resilience scale, and 10-item Perceived Stress Scale were measured.

**Results:**

A total of 2,557 participants were included. Spearman correlation analysis revealed that lower resilience was associated with more somatic symptoms (*p* < 0.001). The conditional process model was proved (indirect effect = −0.01, 95% confidence interval = [−0.015, −0.002]). The interaction effects between perceived stress and sex predicted somatization (*b* = 0.05, *p* = 0.006).

**Conclusion:**

Resilience is a key predictor of somatization. The mediating effects of perceived stress between resilience and somatization work in the context of sex difference. Sex-specific intervention by enhancing resilience is of implication for alleviating somatization during the coronavirus disease 2019 epidemic.

## Introduction

Somatization is common in primary care across cultures (Gureje et al., 1997). Approximately 20% of primary care patients report “non-specific, functional, and somatoform bodily complaints” (Schaefert et al., 2012). A variety of physical symptoms were possible manifestations of somatization, including dizziness (Russo et al., 1994), pains (Asmundson and Katz, 2009), fatigue (Vassend et al., 2018), musculoskeletal complaints (Vassend et al., 2017), and miscellaneous symptoms. People with somatic symptoms always tend to seek medical or non-medical help for reassurance (Zantinge et al., 2005; Budtz-Lilly et al., 2015), but somatization is difficult to treat (Zantinge et al., 2005; Jones and de C Williams, 2019). Moreover, it hinders the understanding of somatization in view of the heterogeneity of somatic symptoms and the difficulty of collecting data from a big sample size within a limited time. Currently, the coronavirus disease 2019 (COVID-19) epidemic has become a public health emergency of international concern (January 31 to February 2, 2020) (Wang et al., 2020), which provided a natural circumstance for a better understanding of epidemic-related somatization during this period. A nationwide survey during the COVID-19 epidemic, covering respondents from 194 cities in China, showed that 5.62% of the respondents reported three physical symptoms, 9.42% reported two physical symptoms, and 15.04% reported one physical symptom (Wang et al., 2020). Therefore, it is of significance to screen risk factors and protective factors for somatization.

Resilience is a dynamic, modifiable factor, and it helps individuals to endure adversities ranging from daily hassles to trauma (Rutter, 1987; Norris et al., 2009; Lehrer et al., 2020). Prior empirical researches have addressed the importance of resilience in the development of somatic symptoms, but the results were inconsistent. The majority of the existing studies are in line with the notion that higher resilience could predict lower levels of somatization (Malarkey et al., 2016; Der Ven Dewsaran-van et al., 2018; Behnke et al., 2019), although very few studies reported different findings (e.g., Um et al., 2014).

Perceived stress is the cognitive appraisal of the objective stressors (Cohen et al., 1983; Hewitt et al., 1992). Recent studies have found that lower levels of perceived stress are associated with higher resilience (Sarrionandia et al., 2018; Smith et al., 2018; Thompson et al., 2018; Sahu et al., 2019). Moreover, it is well known that stress-related etiology is crucial for understanding somatization (e.g., Hewitt et al., 1992; Mischkowski et al., 2019). For instance, perceived stress was a significant predictor of variance across the Symptom Checklist-90 – Revised dimensions in women with systemic lupus erythematosus (Peralta-Ramírez et al., 2018). Notably, stress and physical symptoms may be closely related at multiple levels. A recent review has suggested that both stress and pain are jointly modulated by other psychosocial factors such as beliefs, fears, goals, and the social context (Timmers et al., 2019). Therefore, stressors in the COVID-19 epidemic, such as uncertainty about health (Rothe et al., 2020) and health-related information (Tang et al., 2018; World Health Organization [WHO], 2020 situation report-13), loss of income, social distance, may trigger physical symptoms in a proportion of the general population.

It is worth noting that sex may play an important role in somatization. A study in adolescents found that sex was a moderator in the relationship between the experience of life stress and somatic symptoms (Rehna et al., 2016). A recent study compared three cross-sectional surveys in the general German population in the last four decades and found the prevalence of somatic symptoms was lower in the more recent survey in both men and women, especially in women (Beutel et al., 2020). Therefore, the indirect association between resilience and somatization may also be moderated by sex in Chinese adults.

**FIGURE 1:**
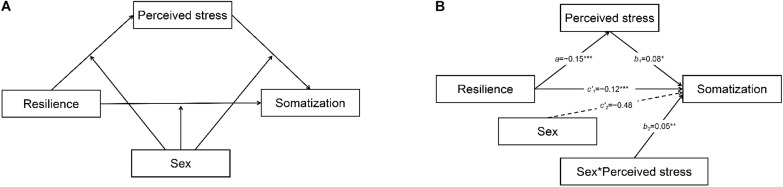
**(A)** Conditional process model of sex on the link between resilience and somatization through perceived stress in conceptual form. **(B)** Conditional process model of sex on the link between resilience and somatization through perceived stress. Dashed lines indicated that statistically insignificant paths between variables. Solid lines indicated that statistically significant paths between variables.

Taken together, no study was investigating the indirect link between resilience and somatization *via* perceived stress. Moreover, the links between resilience, perceived stress, and somatization have not been investigated during an infectious disease epidemic. In this study, we aim to explore if resilience would be negatively associated with somatization in people seeking crisis intervention during the COVID-19 epidemic. Such an association might be mediated by perceived stress, and this mediation model might be moderated by sex. We synthesize our hypotheses in a “conditional process” (or moderated mediation) model, depicted conceptually in Figure 1A. First, the interactive effects of sex were estimated on perceived stress and on somatization. Second, we examined the nature of the moderation effects in the model.

## Materials and Methods

### Participants

This study recruited a total of 4,107 (1,345 males and 2,762 females) participants in 31 provinces of China, and they completed the questionnaires before they started an online self-help crisis intervention. One of the participants was excluded due to too short submission time, and 1,048 of them were excluded due to primary school education or less. Another 15 of them were excluded because they lived outside of China. Moreover, considering that some factors might influence the results of this study, 15 of the participants were excluded because their relatives or friends were infected with COVID-19, and 189 participants were excluded because they have a history of mental disorder or are taking medication. Finally, after dropping 282 questionnaires with high repetition rates in response and scores beyond plus or minus three standard deviations, a sample of 2,557 participants was analyzed. In the remaining sample, there were 1,210 subjects from Guangdong province, 812 subjects from Qinghai, 81 subjects from Beijing, and 57 from Sichuan. In addition, 101 subjects were from Hubei, and 68 of them were from Wuhan City. The other 296 subjects were from other provinces.

### Measures

#### Ten-Item Connor–Davidson Resilience Scale

The Connor–Davidson resilience scale (CD-RISC) measures the ability to recover quickly from stress (Connor and Davidson, 2003). Campbell-Sills and Stein (2007) simplified the original 25 items and retained 10 items reflecting the ability to tolerate challenges such as item 8 (“Tend to bounce back after illness or hardship”). The new 10-item unidimensional scale (CD-RISC) has a good internal consistency (Cronbach’s alpha = 0.85). Every item is rated on a five-point scale (0 = “not true at all” to 4 = “true nearly all of the time”). The Chinese version was modified, and its reliability and validity have been examined in the Chinese population (Yu and Zhang, 2007). In the present study, the Cronbach’s alpha value of 0.95 indicated good reliability.

#### Chinese Version of the Symptom Checklist-90 Somatization Subscale

The Symptom Checklist-90 somatization subscale (SCL-90-SOM) has a good internal consistency (Cronbach’s α = 0.86) to summarize people’s complaints of bodily dysfunction with (Derogatis et al., 1976). It contains 12 items, with each item rated on five points (1 = “not at all” to 5 = “extremely serious”). The Chinese version of SCL-90 was validated and widely used in Chinese mental health research (e.g., Ren, 2009). In the current study, the Cronbach’s α coefficient of the SCL-90-SOM was 0.87. The scores on SCL-90-SOM were applied to index the severity of somatic symptoms in the general population.

#### Ten-Item Perceived Stress Scale

The Perceived Stress Scale (PSS) is a self-report psychometric measure conducted to detect one’s level of perceived stress in terms of unpredictability, lack of control, and overload (Cohen et al., 1983). Each items is scored on a five-point Likert scale (0 = never, 1 = almost never, 2 = sometimes, 3 = fairly often, and 4 = very often). Six of the items evaluated the frequency of negative thoughts (e.g., “how often have you found that you could not cope with all the things that you had to do”), and the remaining items evaluated the frequency of positive thoughts (e.g., “how often have you felt that you were on top of things?”). A total score is calculated by reverse scoring for the four positive items and adding the scores for all items. The Chinese version of the scale has been widely used and demonstrated good reliability and validity (e.g., Ng, 2013). In the current study, Cronbach’s alpha value for this scale was 0.85.

#### Demographic Information

The demographic information included age, sex, height, body weight, education (primary school or less, middle school, high school, etc.), occupation (mainly teachers, students, medical staff), marital status (unmarried, married, widowed, divorced, or remarried), severe acute respiratory syndrome (SARS) experience, annual household incomes, history of chronic illness or psychiatric diagnosis, medication, smoking and drinking status, etc. The participants answered yes or no to a question about the SARS experience (Have you ever experienced the SARS epidemic in person?). The history of chronic illness included chronic illness in the kidney, liver, cardiovascular system, endocrinological system, etc. The history of psychiatric diagnosis was also asked. Besides, in the questions about smoking and drinking status, participants were asked to choose one of three options (yes, has quit, never).

### Procedure

Participants in the COVID-19 crisis intervention were invited online by a WeChat Mini-Program to minimize face-to-face interaction. They were asked to complete demographic information and a set of questionnaires embedded in the WeChat crisis intervention Mini-program. The 7-day self-help intervention was based on a low-intensity psychological intervention, Problem Management Plus (PM+) (Dawson et al., 2015). The main purpose of the intervention, stress reduction, was showed on the webpage. The intervention was designed as 10–20 min per day and invited the participants to complete the courses in 7 consecutive days. Before they started the self-help intervention, participants saw themes of every day, including relaxation, stability, self-efficacy, social support, keeping healthy, hope, and a sense of control. All this information might help them decide whether to complete the questionnaire and start the intervention. The Institutional Review Board of the Institute of Psychology, Chinese Academy of Sciences, approved the carryout of this study. The enrollment of participants was carried out in accordance with the Declaration of Helsinki. Online informed consent was obtained from all participants, and they were guaranteed that their privacy would be protected. Data were collected during the period from April 10 to July 31, 2020, when online interventions were carried out in the general population to help people cope with the COVID-19 outbreak.

### Statistical Analyses

SPSS Statistic v26.0 and the SPSS macro program PROCESS v3.4 created by Hayes were applied in our analyses. First, normal distribution was tested with Kolmogorov–Smirnov test for every variable, and no variable was found to be normally distributed (all *p* < 0.001). Descriptive statistics were computed by sex and occupation for the demographic information and main study variables. Education was calculated according to the academic year required to obtain the degree (e.g., if a participant has obtained a bachelor’s degree, the participant’s education is recorded as 16). Marital status was divided into two categories, married (including married and remarried) or unmarried (including unmarried, divorced, and widowed). Categorical variables, such as marital status and smoking, were expressed using percentages. Continuous variables, such as age and SCL-90-SOM scores, were presented as mean and standard deviation. Second, Spearman correlation analysis was performed among 10-item CD-RISC, PSS, and SCL-90-SOM scores. Third, according to our hypotheses, the current study used a conditional process analysis (Hayes, 2018) to estimate the influences of sex (moderator) and perceived stress (mediator) on the relationship between resilience and somatization. We used ordinary least squares regression and estimated the 95% bias-corrected confidence interval (CI) for conditional indirect effects with 5,000 resampled samples to test the theoretical hypothesis model (Figure 1A). If the 95% CI at different values of the moderator or the difference between the conditional indirect effects of predictor variable at those values does not include zero, it means that statistics are significant (Hayes and Rockwood, 2020). Model 59 was used to test the moderating effect of sex between resilience, perceived stress, and somatization. After controlling for occupation, only the moderating effect of sex between perceived stress and somatization was significant (*b* = 0.05, *p* = 0.012, 95% CI = [0.011, 0.089]). The difference of sex between conditional indirect effects was not significant (index = −0.01, 95% CI = [−0.025, 0.003]). Therefore, Model 14 was used to examine our hypotheses further.

## Results

### Demographic Characteristics

The average age of all participants was 30.56 years (SD = 10.78), and among them, 48.5% had a high school education or less. Besides, 0.5% of the annual income of the family exceeded 1,000,000 RMB, and 49.4% of the family earned less than 80,000 RMB annually. Of the participants, 78.5% reported a body mass index <24 kg/m^2^ (21.51 ± 3.39 kg/m^2^). In addition, 4.5% were smokers, and 7.8% drank alcohol in their daily lives. Sex difference was significant in marital status (*p* < 0.001). According to the results, the results indicated significant sex differences in resilience (*p* < 0.001), perceived stress (*p* < 0.001), and somatic symptoms (*p* < 0.001), with the female having lower resilience and suffering more stress as well as more somatic symptoms (see Table 1). In addition, marital status and annual household incomes were both significantly different among occupations (both *p* < 0.001). The results also indicated significant differences in resilience (*p* = 0.010), perceived stress (*p* < 0.001), and somatization (*p* < 0.001) among teachers, students, medical workers, and other occupations. Multiple comparisons showed that only students had significantly lower resilience than people with other occupations (*p* = 0.042). Students had less somatization than medical workers (*p* < 0.001) and teachers (*p* < 0.001). Medical workers had higher perceived stress than three other types (all *p* < 0.001; see Table 2).

**TABLE 1:** Descriptive statistics and differences of sex for all variables.

	Total	Male	Female	χ^2^ or Z	*p*
			
Variables	(*N* = 2,557)	(*N* = 626)	(*N* = 1,931)		
Age (years)***	30.56 ± 10.78	27.97 ± 12.73	31.40 ± 9.93	−5.58	<0.001
BMI (kg/m^2^)***	21.51 ± 3.39	22.05 ± 4.03	21.34 ± 3.14	−4.04	<0.001
Education (years)***	13.01 ± 3.39	12.24 ± 3.30	13.26 ± 3.39	−6.89	<0.001
Resilience***	28.47 ± 8.27	29.44 ± 8.68	28.15 ± 8.11	−4.11	<0.001
Perceived stress***	15.24 ± 7.47	13.97 ± 7.83	15.66 ± 7.30	−5.06	<0.001
Somatization***	14.76 ± 3.74	14.19 ± 3.48	14.94 ± 3.80	−5.50	<0.001
Marital status***				83.98	<0.001
Married	61.0%	11.1%	49.9%		
Unmarried	39.0%	13.3%	35.6%		
**SARS experienced**				0.04	0.834
Yes	39.8%	9.7%	30.2%		
No	60.2%	14.8%	45.4%		
**Annual household incomes**				0.61	0.895
30,000–80,000 RMB	49.4%	12.2%	37.1%		
80,000–300,000 RMB	44.6%	10.8%	33.8%		
300,000–1,000,000 RMB	5.0%	1.3%	4.3%		
More than 1,000,000 RMB	0.5%	0.2%	0.4%		
History of chronic illness*				4.57	0.033
Yes	13.7%	2.7%	11.0%		
No	86.3%	21.6%	64.7%		
**Smoking**				0.74	0.693
Yes	4.5%	1.3%	3.2%		
Has quit	1.6%	0.4%	1.3%		
Never	93.9%	22.8%	71.0%		
Drinking**				9.89	0.007
Yes	7.8%	2.4%	5.4%		
Has quit	2.8%	1.0%	1.8%		
Never	89.4%	21.1%	68.3%		

*N* = 2,557. Contingency table analyses and Mann–Whitney *U* tests were used to examine differences of sex. All data provided as mean ± SD unless indicated otherwise. SD, standard deviation; Education, calculated by academic year, e.g., “middle school” is 9, “technical secondary school” is 11, “high school” is 12, “junior college” is 15, “undergraduate” is 16, “master” is 19, and “doctor” is 23. χ2, Z, and *p* values have been corrected. *P < 0.05; **P < 0.01; ***P < 0.001.

**TABLE 2:** Descriptive statistics and differences of occupation for all variables.

	Medical workers	Students	Teachers	Others	χ^2^ or H	*p*
			
Variables	(*N* = 54)	(*N* = 604)	(*N* = 683)	(*N* = 1,216)		
Age (years)***	36.20 ± 6.99	16.07 ± 4.09	33.99 ± 8.25	35.58 ± 7.63	1263.44	<0.001
BMI (kg/m^2^)***	22.71 ± 3.35	19.65 ± 3.47	21.66 ± 3.10	22.31 ± 3.15	308.53	<0.001
Education (years)***	16.85 ± 2.03	10.95 ± 3.24	15.99 ± 1.25	12.19 ± 3.08	961.17	<0.001
Resilience**	27.70 ± 6.33	27.97 ± 8.36	28.07 ± 7.84	28.97 ± 8.52	11.40	0.010
Perceived stress***	19.46 ± 5.93	14.62 ± 7.51	15.89 ± 7.11	15.01 ± 7.63	37.80	<0.001
Somatization***	15.89 ± 3.29	14.17 ± 3.64	15.82 ± 4.09	14.40 ± 3.46	128.19	<0.001
Marital status***					1,256.06	<0.001
Married	1.6%	0.2%	18.7%	40.6%		
Unmarried	0.5%	23.5%	8.0%	7.0%		
SARS experienced***					273.39	<0.001
Yes	1.0%	2.7%	14.1%	22.1%		
No	1.1%	20.9%	12.6%	25.5%		
Annual household incomes***					58.70	<0.001
30,000–80,000 RMB	0.4%	12.6%	14.4%	22.0%		
80,000–300,000 RMB	1.4%	9.6%	11.7%	21.9%		
300,000–1,000,000 RMB	0.3%	1.3%	0.6%	3.3%		
More than 1,000,000 RMB	0%	0.1%	0%	0.4%		
History of chronic illness***					44.63	<0.001
Yes	0.5%	1.5%	4.9%	6.8%		
No	1.6%	22.0%	21.9%	40.8%		
**Smoking**					6.63	0.356
Yes	0.2%	0.9%	1.2%	2.2%		
Has quit	0%	0.3%	0.3%	1.0%		
Never	2.0%	22.4%	25.2%	44.3%		
**Drinking**					3.35	0.763
Yes	0.3%	1.8%	2.2%	3.5%		
Has quit	0.1%	0.8%	0.7%	1.3%		
Never	1.8%	21.0%	23.8%	42.8%		

*N* = 2,557. Contingency table analyses and Kruskal–Wallis test were used to examine differences in occupation. All data provided as mean ± SD unless indicated otherwise. SD, standard deviation; Education, calculated by academic year, e.g., “middle school” is 9, “technical secondary school” is 11, “high school” is 12, “junior college” is 15, “undergraduate” is 16, “master” is 19, and “doctor” is 23. χ2, H, and *p*-values have been corrected. *P < 0.05; **P < 0.01; ***P < 0.001.

### Correlations Among Study Variables

Spearman correlation analysis revealed that CD-RISC scores were negatively associated with SCL-90-SOM scores (*r* = −0.33, *p* < 0.001, Bonferroni corrected *p* < 0.01). In addition, a negative association was found between CD-RISC and PSS scores (*r* = −0.20, *p* < 0.001, Bonferroni corrected *p* < 0.01). PSS scores were positively associated with SCL-90-SOM scores (*r* = 0.46, *p* < 0.001, Bonferroni corrected *p* < 0.01).

### Conditional Process Analysis for the Proposed Model

A conditional process model was estimated to test whether the mediating role of perceived stress and the moderating role of sex between resilience and somatization after controlling for occupation. As depicted in Table 3, resilience was significantly negatively correlated with perceived stress (*b* = −0.15, *p* < 0.001), and perceived stress was significantly positively correlated with somatization (*b* = 0.08, *p* = 0.021). The results of the conditional process model indicated that the interaction effect between perceived stress and sex significantly predicted somatization (*b* = 0.05, *p* = 0.006).

**TABLE 3:** Model coefficients for the conditional process model.

	Consequent variables								
	
	M (Perceived stress)					Y (Somatization)			
			
Antecedent variables	*b*	SE	*t*	*p*		*b*	SE	*t*	*p*
X (Resilience) *a*	−0.15	0.02	−8.35	<0.001	*c’_1_*	−0.12	0.01	−15.22	<0.001
M (Perceived stress)	–	–	–	–	*b*_1_	0.08	0.04	2.32	0.021
W (Sex)	–	–	–	–	*c’_2_*	−0.48	0.32	−1.49	0.137
M × W	–	–	–	–	*b*_2_	0.05	0.02	2.75	0.006
Constant	19.80	0.73	27.14	<0.001		16.39	0.64	25.61	<0.001
Covariate (Occupation)	−0.11	0.17	−0.67	0.501		−0.01	0.08	−0.17	0.864
	*R*^2^ = 0.03					*R*^2^ = 0.23			
	*F*(2,2554) = 35.51***					*F*(5,1128) = 154.53***			

*N* = 2557. Regression coefficients are shown in each cell; *P < 0.05; **P < 0.01; ***P < 0.001.

The results showed that the indirect effect of perceived stress in mediating the association between resilience and somatization was −0.02 among male (95% CI = [−0.027, −0.014]) and −0.03 among female (95% CI = [−0.036, −0.020]), but the index of moderated mediation and associated bias-corrected bootstrap confidence intervals indicated the conditional process model still holds (index = −0.01, 95% CI = [−0.015, −0.002]). The bootstrapped 95% CI did not include 0 for the pairwise contrasts between the conditional indirect effects. The statistical significance of this test means that two conditional indirect effects are significantly different in the estimation of values of the moderator (Hayes, 2015). Thus, the results in the present study indicated sex moderated the indirect effect (through perceived stress) of resilience on somatization, as shown in Figure 1B.

**FIGURE 2:**
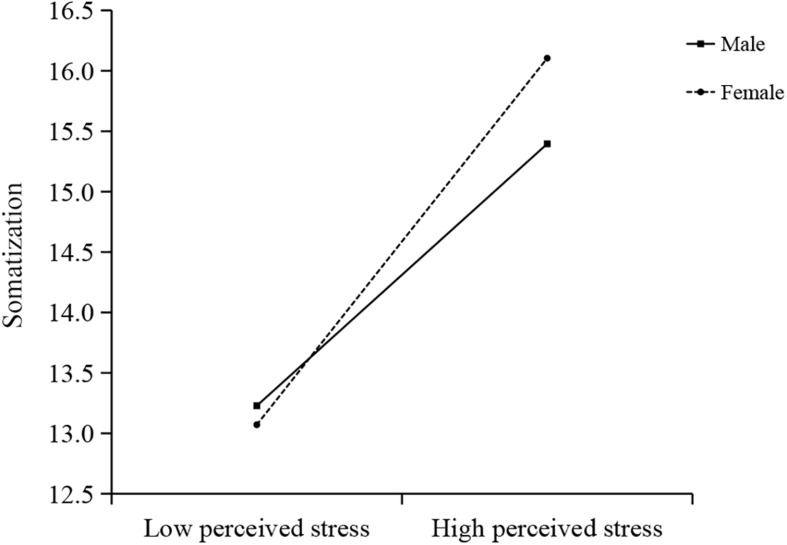
Simple slope analysis showed that sex moderated the relationship between perceived stress and somatization.

Further simple slope analysis in Figure 2 revealed that perceived stress was positively associated with somatization differently in male and female (male: *b* = 0.14, *p* < 0.001; female: *b* = 0.19, *p* < 0.001).

## Discussion

To our best knowledge, this study is the first attempt to evaluate the relationship between resilience and somatization in the context of infectious disease pandemics. We found that lower resilience was associated with higher somatization. Based on a conditional process model, the results showed that the effect of resilience on somatization was moderated by sex and mediated by perceived stress. This indirect relationship was moderated by sex in the second stage of the mediation process. Our findings contribute to understanding the possible sex-specific indirect ways in which resilience influences somatic symptoms through perceived stress.

### Resilience and Somatization

This study revealed that lower resilience was related to higher somatization in participants during COVID-19. This is consistent with some previous studies using different measurements and bearing different cultural backgrounds (Um et al., 2014; Malarkey et al., 2016; Der Ven Dewsaran-van et al., 2018). For example, Malarkey et al. (2016) reviewed recorded symptoms at an outpatient clinic at a United States university, and yielded five clusters of symptoms, which partly overlapped with the SCL-90-SOM items. Although they used another resilience scale different from the current study, negative associations were also found between resilience and the five clusters of symptoms. Recently, some studies found several resilience factors (self-compassion and sense of coherence) were independently associated with less somatic symptoms (Der Ven Dewsaran-van et al., 2018; Behnke et al., 2019). On the contrary, a positive association between resilience and somatization was observed in a Korean sample (Um et al., 2014). It was found that patients who embraced both high depression and high resilience had the highest somatization level compared with those with low depression or low resilience. Um et al. (2014) recruited patients with a diagnosis of depressive disorders, whereas we recruited the general public interested in the crisis intervention during COVID-19. The inconsistency may be attributed to sampling characteristics and sample size in the Korean study or other possible moderators.

Besides, a few studies had suggested positive outcome of intervention on resilience before or after SARS or H1N1 influenza epidemic with various treatments and measurements, whereas somatization was not among the main outcomes concerned (Ng et al., 2006; Maunder et al., 2010; Aiello et al., 2011). To reduce stress and build resilience, Aiello et al. (2011) and his colleagues detected the significant effect of a training session on coping ability among a proportion of participants experiencing the H1N1 pandemic. Similarly, Ng et al. (2006) tried a 1-day body–mind–spirit group debriefing to develop resilience in a Hong Kong community sample of people living with chronic diseases right after the SARS outbreak. The participants reported a significant decrease in depression and negative self-appraisal, which was sustained until the 1-month follow-up. Interestingly, a computer-assisted training course was effective in building resilience in health-care workers well before the H1N1 influenza pandemic (Maunder et al., 2010). Moreover, a recent study suggested that resilience might serve as a stress buffer, as well as a direct determinant of cardiometabolic health (Lehrer et al., 2020). Taken together, improving an individual’s resilience should be considered as an alternative treatment to desomatization in the future, and evaluation of somatization should be designed in the interventions on resilience during or after infectious disease epidemic.

### Moderating Role of Sex

To our best knowledge, this is the first study to explore whether sex will moderate the relationship between perceived stress and somatization in the general population during an infectious disease pandemic. To be specific, the relationship between perceived stress and somatization was stronger in females than in males. However, Ramírez-Maestre and Esteve (2014) only observed the association between fear-avoidance and pain intensity in patients with chronic pain in men. The reason for the existing inconsistent findings may be cultural differences, sex role, recall bias, features of stressors, or perceived social support or emotion regulation strategies during the COVID-19 epidemic, which need more evidence to support (Houtveen and Oei, 2007; Wang et al., 2019).

For the link between resilience and perceived stress, the results showed that the relationship between resilience and perceived stress was not moderated by sex, although we found significant sex differences in both resilience and perceived stress. No concordant results were yielded on sex difference in perceived stress in previous studies (e.g., Thompson et al., 2018; Lehrer et al., 2020). However, several prior pieces of the research reported that resilience showed sex differences in various populations (e.g., Sun and Stewart, 2007; Erdogan et al., 2015; Masood et al., 2016). Sex hormone-related neuropsychological mechanisms are potential explanations to unravel the sex difference in resilience partly. For instance, low psychological resilience was related to compromised control of neural circuits involved in emotion regulation (Southwick and Charney, 2012; Gupta et al., 2017; Liu et al., 2019), and these circuits were influenced by sex hormones (Van Honk and Schutter, 2006; Liu et al., 2019). Furthermore, inconsistent findings were reported about sex differences in the association between resilience and perceived stress. For example, two previous studies found that female medical students reported significantly lower resilience and higher perceived stress compared with males (Rahimi et al., 2014; Thompson et al., 2018). Another study also found that the association between resilience and perceived stress was significant in both female and male young adults, with a stronger interrelationship in females (Yalcin-Siedentopf et al., 2020). However, a study reported that trait resilience mediated the association of childhood maltreatment with perceived stress in young female adults, whereas no significant mediating effects were found in males (Hong et al., 2018). The COVID-19 epidemic and the specific population might contribute to these inconsistencies between the findings of previous studies and the current study.

### Limitations

This study has several limitations. First, the observational nature and the cross-sectional design limit the interpretability of the mediation analysis. A longitudinal study with the same sample should be conducted to detect the causal link between resilience and somatization with the development of infectious disease epidemics. Second, this self-selected sample was obtained from the population consisted of people who were intended to use online self-help intervention, so our findings might not be suitable for the general population. Third, self-reported physical symptoms may not always be as reliable as the assessment by professionals. Symptom reports in people with somatic symptoms might increase as time passed by, and the reason might be a shift from episodic knowledge to semantic beliefs (Houtveen and Oei, 2007). Fourth, we did not consider whether some participants experienced childhood trauma before, as traumatic stress was also reported to foster the development of somatization (Berger et al., 2014). Fifth, female sample constitutes the majority of this study, and significant sex differences are found in age, body mass index, education, marital status, and drinking. We carried out multiple regression analyses and found that none of these variables are predictive of somatization in males or females.

## Conclusion

Resilience is a key predictor of somatization. Sex differences should be noticed in the associations among resilience, perceived stress, and somatization. The findings in the current study have important implications on crisis intervention during and after the COVID-19 epidemic. First, promoting resilience should be included as the main purpose in crisis intervention. Because resilience is a multidimensional construct with various measurements, the related treatment components and measurements should be chosen with intention. Second, coping strategies on somatization may be delivered in a sex-specific way. Third, cultural sensitive tools for resilience should be considered in the future studies and clinical interventions.

## Data Availability Statement

The raw data supporting the conclusions of this article will be made available by the authors, without undue reservation.

## Ethics Statement

The studies involving human participants were reviewed and approved by the Institutional Review Board of Institute of Psychology, Chinese Academy of Sciences. The patients/participants provided their written informed consent to participate in this study.

## Author Contributions

FS: conceptualization, formal analysis, methodology, writing – original draft, and writing – review and editing. CZo: data curation, formal analysis, and writing – original draft. WQ: data curation. CZa: writing – original draft. ZL: conceptualization, methodology, project administration, and investigation. XZ: investigation and writing – review and editing. All authors contributed to the article and approved the submitted version.

## Conflict of Interest

The authors declare that the research was conducted in the absence of any commercial or financial relationships that could be construed as a potential conflict of interest.
